# Neuronal Trafficking of the Amyloid Precursor Protein—What Do We Really Know?

**DOI:** 10.3390/biomedicines9070801

**Published:** 2021-07-10

**Authors:** Tong Lin, Lars O. Tjernberg, Sophia Schedin-Weiss

**Affiliations:** Division of Neurogeriatrics, Department of Neurobiology, Care Sciences and Society, Karolinska Institutet, 17164 Stockholm, Sweden; tong.lin@ki.se

**Keywords:** Alzheimer’s disease, amyloid precursor protein, amyloid β-peptide neuronal transport, sorting-related receptor with A-type repeats, sortilin

## Abstract

Alzheimer’s disease (AD) is the most common type of dementia, contributing to 60–80% of cases. It is a neurodegenerative disease that usually starts symptomless in the first two to three decades and then propagates into a long-term, irreversible disease, resulting in the progressive loss of memory, reasoning, abstraction and language capabilities. It is a complex disease, involving a large number of entangled players, and there is no effective treatment to cure it or alter its progressive course. Therefore, a thorough understanding of the disease pathology and an early diagnosis are both necessary. AD has two significant pathological hallmarks: extracellular senile plaques composed of amyloid β-peptide (Aβ) and intracellular neurofibrillary tangles composed of hyperphosphorylated tau protein, and the aggregation of Aβ, which starts in earlier stages, is usually claimed to be the primary cause of AD. Secretases that cleave Aβ precursor protein (APP) and produce neurotoxic Aβ reside in distinct organelles of the cell, and current concepts suggest that APP moves between distinct intracellular compartments. Obviously, APP transport and processing are intimately related processes that cannot be dissociated from each other, and, thus, how and where APP is transported determines its processing fate. In this review, we summarize critical mechanisms underlying neuronal APP transport, which we divide into separate parts: (1) secretory pathways and (2) endocytic and autophagic pathways. We also include two lipoprotein receptors that play essential roles in APP transport: sorting-related receptor with A-type repeats and sortilin. Moreover, we consider here some major disruptions in the neuronal transport of APP that contribute to AD physiology and pathology. Lastly, we discuss current methods and technical difficulties in the studies of APP transport.

## 1. Alzheimer’s Disease (AD)

A healthy adult brain contains about 100 billion neurons, and each of these neurons has long, branching extensions that form about 100 trillion functional synapses enabling signal transmission between individual neurons [1]. In neurodegenerative disorders, for example, AD that contributes to 60–80% of dementia cases, synapses are lost, and neurons gradually deteriorate [1]. There are over 35 million affected individuals worldwide, and the total number of people with AD is projected to reach 100 million in 2050 [2,3]. It is mainly present in the elderly population with a 1 in 10 prevalence in people over 65, and the risk of obtaining AD doubles every 5th year between the age of 65 and 90 [4]. The disease usually starts symptomless in the first two to three decades and then propagates into a long-term, irreversible disease that results in the progressive loss of memory, reasoning, abstraction and language capabilities. It is a complex disease, involving a large number of entangled players, and there is no effective treatment so far to cure it or alter its progressive course. Therefore, a thorough understanding of the disease pathology and an early diagnosis are both necessary.

One of the two significant pathological hallmarks of AD is extracellular senile plaques composed of amyloid β-peptide (Aβ), and this aggregation of Aβ starts in earlier stages. There are also reported familial cases with early onset due to mutations in amyloid precursor protein (APP) or APP processing genes. In this review, we thus focus on APP. According to the amyloid hypothesis, the aggregation of Aβ is the primary cause of AD; this accumulation is toxic and thus lethal to neurons, leading to chronic inflammation, neurodegeneration and the loss of neuronal functions and synaptic connections. Another characteristic of AD is tangled neurons caused by hyperphosphorylated, aggregated forms of tau protein [5]. There are many investigations ongoing to determine the connections between Aβ and tau pathology, and it has been suggested that APP induces intracellular tau aggregation and that the extracellular domain of APP is required for this to occur [6,7]. Therefore, to understand AD pathogenesis and progression, it is essential to understand where APP is located and how it is transported within neurons.

## 2. Amyloid Precursor Protein

**Figure 1:**
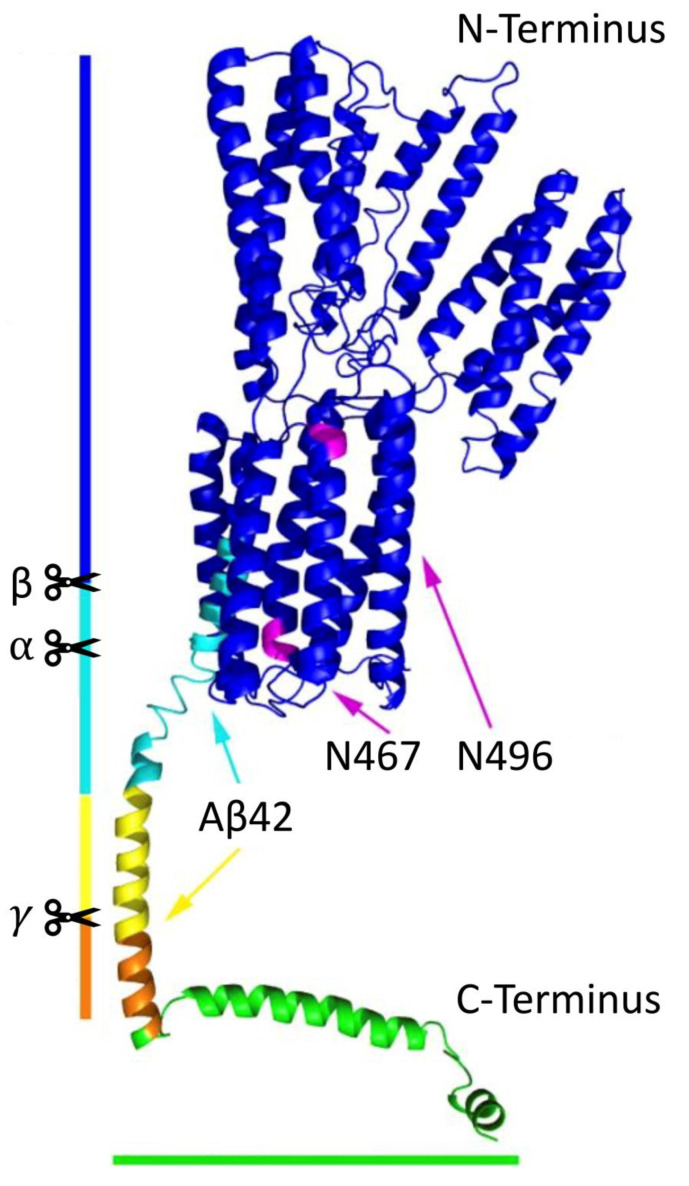
3D protein structure of APP695. Blue, magenta and turquoise indicate the extracellular domain towards its N-terminus; orange and yellow indicate the transmembrane domain; green indicates the intracellular domain towards its C-terminus; turquoise and yellow indicate Aβ42; magenta indicates two N-glycosylation sites (N467 and N496). Black scissors indicate the cleavage sites of three major secretases (⍺-, β- and 𝛾-secretase) on APP. This 3D protein structure was produced by I-TASSER and visualized by the PyMol Molecular Graphics System, Version 2.5.0, Schrodinger, LLC, Crayon, Solna, Sweden.

**Figure 2:**
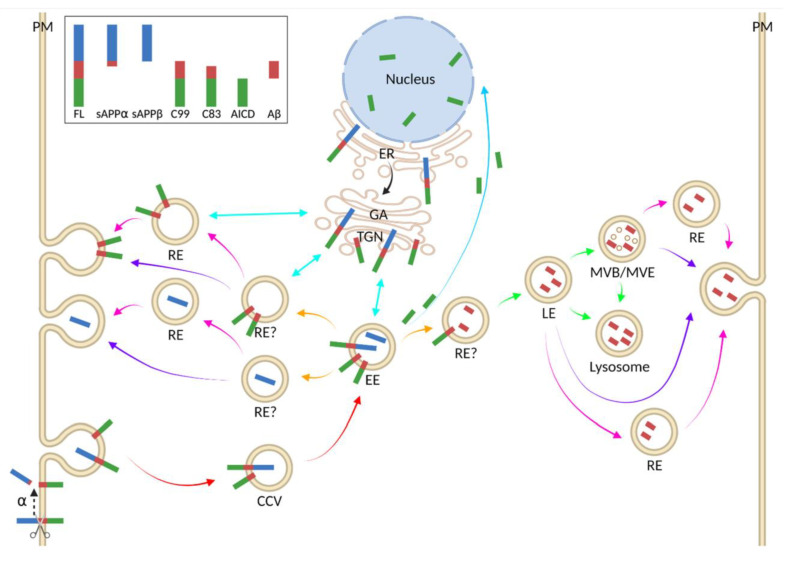
Distribution of APP in neurons. APP traffics along secretory, endocytic and recycling routes and its cleavages occur at multiple sites. ER is short for endoplasmic reticulum, GA is short for Golgi apparatus, TGN is short for *trans*-Golgi network, EE is short for early endosome, LE is short for late endosome, RE is short for recycling endosome, MVB is short for multivesicular body, MVE is short for multivesicular endosome, CCV is short for clathrin-coated vesicle. Blue, green and red bars represent full-length APP (FL); blue (and red) bars represent N-terminal fragments (NTFs; including sAPP⍺ and sAPPβ); red and green bars represent C-terminal fragments (CTFs; including C83, C99 and AICD); and red bars represent Aβs. The black arrow indicates the trafficking of APP from the ER to the GA/TGN; turquoise arrows indicate the trafficking of APP between endocytic compartments and the TGN; red arrows indicate the endocytosis of APP from the PM (the scissor indicates the major ⍺-secretase cleavage site of APP, where membrane-bound full-length APP becomes C83); magenta and purple arrows indicate the indirect (via REs) and direct recycling of APP towards the PM and secretion into the extracellular space; green arrows indicate the trafficking of APP within the endocytic pathway towards the lysosome; orange arrows indicate the potential sorting of APP in EEs; and the plain blue arrow indicates the trafficking of AICD towards the nucleus. This figure was produced with BioRender.com.

APP, a ubiquitously expressed type I transmembrane glycoprotein, contains a short C-terminal intracellular domain and a large highly preserved extracellular domain (Figure 1). It belongs to the APP protein family, of which there are also two other homolog proteins that share this conserved structure (except for the Aβ domain): APP-like proteins 1 and 2 [8]. APP is located at chromosome 21, and it undergoes alternative splicing of 18 exons to eventually generate three major protein isoforms, of which APP695 (695 amino acids long) is the most abundant in the brain. APP and its processed products are essential for normal neuronal development. For example, Aβ [9] and soluble APP⍺ (sAPP⍺) [10] both contribute to various physiological activities; APP intracellular domain (AICD) possibly acts as a cofactor for transcriptional activation in the nucleus with an assisted transport from the adaptor protein Fe56 (Figure 2, plain blue arrow) [11], and it is suggested to be a key regulator in the formation, maturation and function of neuromuscular synapses [12]. 

APP can be found at the plasma membrane (PM) and the endomembrane of transport vesicles, where it is processed sequentially by two different transmembrane aspartyl proteases [7]. The first cleavage is mainly performed either by ⍺- or β-secretases. In the non-amyloidogenic ⍺-secretase pathway, it is cleaved by a membrane-bound zinc metalloproteinase (mainly a disintegrin and metalloproteinase-domain-containing protein 10/ADAM10) between residues Lysine 16 and Leucine 17 in the extracellular region to produce membrane-bound C83/C-terminal fragment ⍺ (CTF⍺) and sAPP⍺. In the amyloidogenic pathway, on the other hand, APP is cleaved by a type I membrane-bound aspartyl protease, denoted “β-secretase” (mainly β-site APP-cleaving enzyme 1/BACE 1), to produce membrane-bound C99/CTFβ and soluble APPβ (sAPPβ). This cleavage results in a CTF containing the Aβ sequence within its transmembrane and extracellular/lumenal domains. Amyloidogenic cleavage is favored as a result of the elevated retention of APP in acidic compartments and a decrease in its internalization from the PM results in enhanced non-amyloidogenic processing [13]. The resulting CTF⍺ and CTFβ are then further processed by γ-secretase [14], an enzyme complex that is composed of 4 subunits: presenilin 1 or 2, which functions as the catalytic core of the enzyme complex; presenilin enhancer 2, which stabilizes the mature enzyme complex; anterior pharynx-defective 1, which functions in the assembly of the enzyme complex; and nicastrin, which functions in substrate recognition; the loss of any subunit abolishes its proteolytic activity [15]. The cleavage by γ-secretase is performed within the transmembrane domain of CTFs to produce AICD together with either p3 (in the non-amyloidogenic pathway) or Aβ of different lengths (in the amyloidogenic pathway) [7]. The most pathological, aggregation-prone and neurotoxic Aβs are isoforms with 42 or 43 amino acids (Aβ42 or Aβ43) [16,17]. These species can polymerize into toxic aggregates and are significantly increased in AD brains [17,18]. Overall, APP and its processed products are dynamically transported within distinct vesicular populations, wherein the key steps of APP processing occur (Figure 2). Thus, alterations in APP transport can directly influence whether APP releases a nontoxic peptide or the neurotoxic Aβ.

## 3. Cellular Transportation System

### 3.1. Secretory Pathway

Secretion is the fundamental physiological process to deliver soluble proteins and cargo to the extracellular space for a number of purposes, such as cell growth, cell homeostasis, cytokinesis, hormone release and neurotransmission. There are two types of secretion: (1) constitutive secretion, a constantly ongoing process present in every cell, and (2) regulated secretion, which requires an extracellular stimulus and involves receptor-mediated transport. 

The conventional secretory pathway transports secretory proteins from the endoplasmic reticulum (ER), through the Golgi apparatus (GA)/*trans*-Golgi network (TGN), to the PM or extracellular space via secretory vesicles or granules [19]. Proteins to be sorted into this pathway usually require a signal peptide, an N-terminal extension of newly synthesized proteins that mediates protein targeting and is later removed by signal peptidases. Firstly, newly synthesized non-ER-resident proteins exit at the ER-exit sites (also known as transitional elements or transitional ER) where coat protein complex (COP) II-coated vesicles are assembled (known as COPII-mediated transport). Then, these ER-derived cargoes enter the lumen of *cis*-Golgi at the Golgi arrival sites and travel through stable cisternae of the GA within COPI-coated vesicles (known as anterograde COPI-mediated transport), which are also responsible for the (retrograde) intra-Golgi retrieval of integral Golgi proteins from older to younger cisternae, as well as the recycling of materials back to the ER [20]. These cisternae of the GA continuously mature from *cis*-to-*trans*, during which the fusion of COPII vesicles at the *cis* side of the GA gives rise to newly formed *cis*-cisternae while *trans*-most cisternae mature into the TGN. The TGN is therefore the central hub where cargo is sorted into secretory vesicles or granules. These cargo-containing vesicles or granules bud from a donor membrane and eventually fuse with an acceptor membrane for delivery towards two different destinations: (1) the PM and extracellular space or (2) the endocytic and autophagic pathways. These vesicles or granules bind to and travel along the cytoskeleton across the cytoplasm with assistance from specific scaffolding or adaptor or effector proteins that directly or indirectly tether the cytoplasmic tail of sorting receptors upon recognition of sorting signals. For those destined to the PM, soluble proteins are then discharged into the extracellular space via exocytosis, while integral proteins remain in the PM. Other newly synthesized proteins that are destined for residence within organelles of secretory, endocytic and autophagic pathways move from the ER to their final destinations in a similar manner.

The unconventional secretory pathway, on the other hand, is responsible for the delivery of both cytoplasmic and integral membrane proteins lacking a signal peptide to the extracellular space. One example is secretory autophagy (also known as autosecretion) [21]. Regulated autophagy, unlike constitutive autophagy, is initiated by an inhibition of the mTOR1 complex that activates a cascade of phosphorylation events and thus leads to the formation of a cup-shaped membrane in the vicinity of its targeted cargo at the phagosome assembly site. This isolated membrane (known as a phagophore) then gradually wraps around the cargo (such as misfolded proteins, protein aggregates, damaged organelles) to form an autophagosome with the assistance of the autophagy protein family members and microtubule-associated proteins 1A/1B light chain 3 (LC3). Membrane-bound cargo within this secretory autophagy pathway can then be delivered directly or indirectly, via MVBs/MVEs to form amphisomes, to their destined location, where they are released. For example, those destined to the PM are released after an MVB-PM fusion event.

### 3.2. Endocytic and Autophagic Pathways

The primary function of endocytic pathways is to take up, process, degrade and dispose of materials in the cell. It consists of several membrane and vesicular compartments that are able to exchange and transport cargo along the cytoskeleton by controlled fusion and fission events between each other [22,23].

Early endosomes (EEs) are formed by endocytosis, an invagination of a certain portion of the PM. One of the most important endocytic processes occurs in a specific manner that requires a start signal. Clathrin-mediated endocytosis (also known as receptor-mediated endocytosis), for example, can be triggered by the specific binding of extracellular molecules to their membrane-bound receptors. Once this cellular process is triggered, an area of PM (the donor membrane; also known as clathrin-coated pits) starts to deform through the characteristic coat protein clathrin on the inner surface of the PM. This deformation of the PM then buds off towards the cytoplasm and forms a small vesicle (around 100 nm in diameter) around the extracellular cargo. The resulting EEs function as sorting organelles in which endocytosed cargo dissociate from its receptors in a mildly acidic pH environment and is then sorted into distinct downstream pathways. 

There is bidirectional movement between the TGN and endosomes. In both directions, it is a complicated process assisted by a broad range of accessory proteins, among which some are cargo-specific. In anterograde transport, newly synthesized proteins are delivered through secretory pathways to endocytic compartments. During this part, EEs can transform into late endosomes (LEs) or intermediate multivesicular bodies (MVBs)/multivesicular endosomes (MVEs) if they undergo another membrane invagination and fission event to form intraluminal vesicles. From here, EEs or EE-derived LEs/MVBs/MVEs can go into separate pathways: (1) they can recycle the endocytosed portion of the PM and membrane-bound receptors back to their origin and transport soluble proteins into the extracellular space (intraluminal vesicles released as exosomes). This transport toward the PM can be divided into a fast, direct pathway and a slower pathway that goes via recycling endosomes (REs); (2) they can direct cargo to a lysosomal pathway, where it fuses with lysosomes and releases its contents into newly formed endolysosomes for degradation after a series of maturation and fusion events. These LEs are acidic compartments that mediate the final sorting events of cargo prior to its delivery to lysosomes for degradation. LEs, and sometimes also late-late endosomes, share a number of features and subcellular markers with the lysosomes they finally fused with. 

The anterograde flow of cargo is counterbalanced by an influx that follows various retrograde transport pathways to the TGN. EEs and REs containing cargo in this direction go through retrograde transport towards the TGN in the presence of a pentameric retromer adaptor complex [24]. Some of the cargo in this retrograde direction leave *cis*-Golgi at the Golgi exit sites, where cargo-containing COPI vesicles assemble, and end up in the ER, entering at the ER arrival sites. The most common retromer complex here consists of five subunits, a trimer composed of vacuolar protein sorting (Vps) proteins: Vps26-Vps35-Vps29 and a dimer composed of sorting nexins (SNX): SNX1-SNX5, SNX1-SNX6, SNX2-SNX5 or SNX2-SNX6. The trimer in this complex remains attached to endosomes due to its interaction with GTPase Rab5 or Rab7 and forms a highly conserved cargo-selecting subcomplex with the banana-shaped protein Vps35. This Vps35 subunit, serving as the main cargo-binding subunit, then interacts with the Vps26 subunit via its N-terminus and with the Vps29 subunit via its C-terminus. The dimer, on the other hand, has multiple roles: (1) the Bin/Amphiphysin/Rvs domain mediates a membrane interaction to sense membrane curvature and to initiate membrane folding; (2) the phosphoinositide-binding phox homology domain targets phosphatidylinositol 3-phosphate enriched in EEs; (3) the membrane-binding domains interact directly with the endocytic membrane. 

The lysosome is the final compartment within this pathway. It has an acidic environment (pH = 4.5) that is optimal for the activity of hydrolytic enzymes. These enzymes assist in major lysosomal functions to break down materials (such as misfolded proteins) into simple compounds (such as amino acids). The resulting compounds can be recycled back to the cytosol as new cell building materials or transported to other organelles for further use.

An alternative to transport cytosolic proteins and organelles is degradative autophagy (also known as macroautophagy) [21,25]. This is also a lysosome-dependent catabolic pathway involving the elimination of damaged organelles and misfolded proteins. There are two modes of degradative autophagy: one requires a specific binding of cargo onto its receptors, while the other is simply the bulk engulfment of surrounding materials. Double-membrane autophagosomes with sequestered cargo are formed in a similar manner to those in the unconventional secretory pathway, which can then fuse with lysosomes to release their contents into newly formed autolysosomes, with or without a formation of amphisomes with LEs/MVBs/MVEs.

Overall, protein sorting to its destined location in subcellular compartments is essential for proper cell function, and alterations result in cellular dysfunction and diseases. The cellular transportation system, especially endocytic and autophagic pathways, is highly dynamic, with a huge number of participants. Many of the intracellular compartments involved mature gradually through processes of fusion and fission events and thus, share a number of subcellular markers and cannot be considered discrete compartments.

## 4. Lipoprotein Receptors in Cellular Transportation System

### 4.1. Essential Lipoprotein Receptors

The indispensable nature of lipoprotein receptors to gather nutrients, to clear materials and to mediate intracellular transport and signaling brings them to the spotlight of the cellular transportation system [26]. The main endocytic receptors for the uptake of lipid-loaded lipoproteins belong to the low-density lipoprotein receptor (LDLR) family, including the sorting-related receptor with A-type repeats (SorLA). Its family members are type I membrane receptors containing (1) an intracellular domain with at least one NPxY motif (Asn-Pro-X-Tyr) that functions in protein interaction, signal transduction and endocytosis and (2) an N-terminal extracellular domain composed of ligand binding domains (also known as complement type repeats) that confer ligand specificity and epidermal-growth-factor-homology domains that participate in the pH-dependent release of bound ligands after endocytosis. The activity of these lipoprotein receptors assures the proper function of cells, and dysfunction has been reported to be inextricably linked to common human malignancies. Another class of type I receptors is Vps10p-domain receptors that are expressed in neurons [27]. They are multifunctional proteins and share the Vps10p domain for protein interactions but contain additional N-terminal protein binding motifs in some members. They participate as regulators of neuronal viability and function through the regulation of protein transport and signal transduction. They also function as sorting proteins that target a range of ligands, during which these receptors bind to their ligands and shuttle them to destined locations. Loss of activities can thus contribute to the pathophysiology of disorders of the nervous system; for example, SorLA with a gene variant of *SORL1* in AD patients is now considered a major AD risk factor [28,29].

### 4.2. SorLA (Also Known as LR11 and SORL1)

SorLA is predominantly expressed in the TGN and EEs in the neurons of the cortex, hippocampus and cerebellum [30]. It is a Vps10p-domain receptor and a key regulator of intracellular protein sorting, acting as a sorting receptor between the TGN, the PM and endosomes to direct cargo to its correct locations within neurons [31]. It shares a structural similarity with LDLR family members, and it is the only member with a Vps10p domain and six fibronectin-type III domains. The Vps10p domain at its extracellular/lumenal tail serves as a major site for ligand binding, and this binding is lost at low pH (<5.5), suggesting that its receptor–ligand interaction is disrupted in endocytic compartments with a more acidic environment. The fibronectin-type III domains are also thought to be involved in protein–protein interactions.

### 4.3. Sortilin (Also Known as SORT1 and gp95)

Sortilin is also a member of the Vps10p-domain receptor family. It contains a single Vps10p domain for protein interaction that confers a simpler ligand recognition profile compared to SorLA. Based on quantitative RT-PCR from C57BL/6j mice brains and Western blot analysis of astrocytes and hippocampal neurons, it has been found that sortilin is expressed at high levels in neurons [32] and that it is distributed intracellularly in the soma and dendrites of the cortex and hippocampus [33]. 

## 5. Neuronal Transport of APP

### 5.1. Neuronal Transport

Neurons are polarized cells with extreme geometries: one axon and multiple dendrites generally emerge from a single cell body, each of which establishes synaptic contacts with its partners. In a single neuron of the human central nervous system, for example, an axon can contain hundreds to over one million presynaptic sites and a total length of up to over 100 m [34]. This enormous size and complex functional architecture of neurons depend on the intense and exquisitely regulated transport of materials, including synaptic vesicle precursors and mitochondria, to different subcellular compartments, and efficient proteostasis to accommodate a dynamic microenvironment. Therefore, the cellular transportation system is specialized in neurons to ensure the maintenance of presynaptic homeostasis by replenishing presynapses with fresh or additional proteins or organelles and by removing old and defective synaptic components [34]. The complexity of this system is demonstrated by an enormous number of entangled players and their specificity, which overall creates a complicated system that enables a precise delivery of an amount of critical cargo to specific sites [35]. 

There are two types of neuronal transport based on the distance a cargo travels: (1) the localized delivery of presynaptic components to thousands of branches and hundreds of thousands of synapses with extremely high precision and (2) the long-distance delivery between distal regions and the cell center, which requires effective navigation. In both cases, cargo travels along the cytoskeleton that serves to provide tracks for vesicle transport. Two major cytoskeletons involved in neuronal transport are microtubules and actin filaments. In general, microtubules are the major cytoskeleton responsible for long-distance delivery, while actin filament is for short-distance local delivery. For example, secretory vesicles mature as they are actively transported along microtubules toward the actin network at the cell periphery [36]. However, microtubules and actin filaments tend to work collaboratively to achieve complex transport tasks. For example, microtubules are localized close to actin filaments, and the inhibition of microtubule polymerization affects actin coat formation and polymerization on fused vesicles [37]. The dynamics of microtubules can also contribute to how cargo travels: long microtubules support the fast transport of vesicles, while discontinuous ones promote cargo pausing and local delivery in, for example, synaptic areas. Moreover, microtubules continuously self-renew without interrupting active transport and provide a stable platform for neuronal transport and, despite their more complicated bipolar distribution in dendrites, their uniformly polarized network in axons guide the directionality of cargo transport. This navigation of cargo traveling along microtubules is mainly governed by two cytoplasmic microtubule-associated motor proteins: dynein for retrograde transport and kinesin for anterograde transport. During retrograde transport from both dendrites and axons, cargo-containing endosomes mature and fuse with lysosomes on their way to the soma [22,38]. There is also reported bidirectional movement within certain restricted areas in distal neurites and a potential opportunity at some point to exit and proceed to a unidirectional movement [39]. During anterograde transport, on the other hand, various membrane-bound organelle cargoes are transported from their sites of synthesis and packaging in the soma to their final sites of utilization within axons and dendrites. For example, soma-derived lysosomes can be constantly delivered to distal axons to maintain local degradation capacity [40]. Both motor proteins here are normally locked in an auto-inhibition state(s). In order to be activated, kinesins and dyneins must firstly be engaged onto microtubules via electrostatic interaction between positive charges on motor proteins and negative charges on microtubules and undergo conformational changes. Since dyneins work more effectively in teams, there are normally multiple dyneins and 1–2 kinesins simultaneously associated with a single cargo. Although there is an implication of potential competition between the two motor proteins, it has been reported that there is a complicated regulatory system(s) to govern the directed transport of cargo-carrying opposing motor proteins, that is, to determine which motor protein is activated and in which direction cargo travels [41,42]. For example, kinesins and dyneins are coordinately regulated by scaffolding proteins, such that only a single motor type is active at any given time. An example is c-Jun N-terminal kinase-interacting protein 1 (JIP1), which activates the motor activity of kinesin by binding to kinesin heavy chain in a phosphorylation-dependent manner, while its C-terminal tail remains bound to kinesin light chain, or alternatively activates dynein by binding to its p150 domain of the dynactin–dynein complex [43]. 

Neuronal transport can also be categorized based on the type of cargo and its speed: fast transport (>0.5 µm/s) for synaptic vesicle precursors, mitochondria and endocytic and autophagic compartments; slow transport (<0.1 µm/s) for soluble proteins and RNAs [34]. In most cases, the neuronal transport of distinct cargo requires motor protein-cargo selectivity and motor protein-adaptor protein-cargo specificity [44]. Therefore, neuronal transport requires an interplay of several regulatory mechanisms functioning at multiple levels to direct how and when motor proteins initiate, pause, resume and terminate transport and how they respond to local cues to deliver cargo with high precision [45]. 

### 5.2. Neuronal Transport of APP Species

#### 5.2.1. Newly Synthesized APP in the ER Is Delivered to the GA/TGN for Further Sorting via COPII-Coated Vesicles

APP is translated and co-translationally modified in the ER, sorted and packed into COPII-coated vesicles and post-translationally modified and transported through the GA/TGN. Newly synthesized APP is then transported to the GA/TGN for further protein processing and maturation (Figure 2, black arrow). The acidic pH in the TGN or the late GA (pH = 6.0–6.5) is optimal for the activity of many processing enzymes (such as BACE1), suggesting that further processing of APP could also occur in the TGN. A functional disruption of the GA/TGN in primary human neurons leads to an abnormal or incomplete maturation and the intracellular accumulation of newly synthesized APP, which in turn inhibits further the processing and secretion of APP [46]. This functional disruption is done through a brefeldin A treatment that disaggregates the network of GA/TGN from each other and from the ER and thus, retains newly synthesized and immature proteins in the ER; however, this harsh treatment will also result in the disturbed transport of other molecules in neurons. The TGN is thought to be a sorting station for APP. It has been reported that GA-derived APP-containing vesicles show a perinuclear distribution in mouse hippocampal neurons, and an inhibited exit by lowering the temperature from 37 ℃ to 20 ℃ results in a retention of these vesicles, suggesting that somatic APP is sorted in the GA/TGN and transported towards different pathways [47]. Thus, the ER and GA/TGN are two essential sites for the proper production of mature and functional APP.

#### 5.2.2. APP Is Shuttled between the PM, the GA/TGN and Endosomes

There is bidirectional transport between the GA/TGN and endosomes (Figure 2, turquoise arrows). It has been reported that SorLA is primarily found in the soma of mouse hippocampal neurons [32]. The crystal structure of the Vps10 domain in SorLA indicates a potential binding site for APP species and a peptide-binding-induced conformational rearrangement of this domain, suggesting APP–SorLA interaction [48]. SorLA is continuously shuttled between the TGN and endosomes, acting as a sorting receptor. It interacts functionally with adaptor proteins (Golgi-localized, 𝛾-adaptin ear-containing, ADP-ribosylation factor-binding proteins/GGAs for anterograde transport and retromer complex for retrograde transport) and its C-terminal tail consisting of ligand binding sites is crucial for adaptor protein binding and SorLA-dependent sorting [49]. The retromer complex is highly colocalized with the TGN and EEs in mouse hippocampal neurons, supporting its major transport tasks between the TGN and EEs [50]. It has been reported that disruptions in GGA or retromer complex binding to SorLA in mouse hippocampal neurons lead to defects in APP anterograde or retrograde transport [51]. Some studies show that an interruption of retromer-mediated APP recycling to the TGN could result in an accumulation of endocytosed APP in EEs and a reduction of APP processing by an increase in the colocalization between significantly enlarged EEs and APP species in Vps35-deficient mouse hippocampal neurons [50], which could provide an indication of a shift in the APP subcellular location towards EEs in the absence of retromer complex. However, other researchers report that EEs shuttle internalized APP back to the TGN and thus, decrease the production of Aβ [30]. This idea is supported by experiments showing that the co-expression of SorLA and APP in mouse cortical neurons leads to a significant increase in APP retrograde transport and a significant decrease in stationary vesicles containing either APP or SorLA, suggesting that SorLA induces the mobilization of APP retrograde transport [52]. Interestingly, APP dimerization causes a significant reduction of APP/SorLA co-transport, indicating a low affinity of SorLA towards APP dimers [52].

APP species delivered to the PM can also be endocytosed into EEs (Figure 2, red arrows), which largely occurs by clathrin-mediated endocytosis through interactions between the internalization motif YENPTY in APP (residues 682–687 of APP695), clathrin, adaptor protein 2 and cytoplasmic adaptor protein Fe65 (also known APBB1) [7,53]. This notion is supported by stimulated emission depletion (STED) microscopy studies showing that full-length APP and/or its CTFs are present in clathrin-coated pits in the PM and in the endomembrane of clathrin-coated vesicles of mouse hippocampal neurons [46]. Moreover, disruption in the YENPTY motif results in inhibited APP endocytosis in mouse hippocampal neurons [47]. This could be due to a disrupted interaction with adaptor proteins caused by a mutation within the YENPTY motif, and thus contributes to a reduced complex formation [53]. APP endocytosis is also cholesterol-dependent, and the depletion of flotillin-2 in rat hippocampal neurons resulted in a significant reduction of APP internalization [54]. APP is therefore suggested to be clustered in cholesterol-rich coated pits before it is sent into a specialized clathrin-dependent endocytic pathway that requires a specific subset of adaptor proteins [54]. Moreover, surface-labeled APP species can be internalized into mouse cortical neurons through actin-dependent macropinocytosis and eventually colocalize with lysosomes, through which macropinosomes are known to rapidly fuse [55].

APP species can be recycled back to the PM directly or indirectly (Figure 2, purple and magenta arrows). The direct or fast recycling of endosomes involves a retromer complex similar to the one that directs APP from EEs to the TGN, while the indirect or slow recycling requires REs. It has been shown in mouse hippocampal neurons that APP can be rerouted to TfR-positive REs, where BACE1 resides [47]. The acidic pH of REs (pH = 6.0–6.5), optimal for BACE1 activity, is confirmed by a rapid quenching of its fluorescent signal in the pHluorin:APP construct.

#### 5.2.3. APP Is Directed to Lysosomes for Degradation

Finally, APP species can be delivered to lysosomes for degradation (Figure 2, green arrows), and lysosomal dysfunction causes a delayed or inefficient degradation and thus, an accumulation of CTFs in mouse cortical neurons and a possible activation of alternative clearance via exosomes [56]. This delivery of materials is mostly from axons/dendrites towards the soma, during which endosomes undergo a series of fusion and maturation steps to acquire lysosomal properties, including hydrolytic enzymes and highly acidic lumen (pH = 4.5). For example, there is a spatial heterogeneity of endolysosomal compartments in the dendrites of mouse cortical [57] and hippocampal neurons [58]: EEA1- and Rab7-positive endosomes are found throughout dendrites; early LEs (Rab7^+^/LAMP1^−^) are abundant in medial and distal dendrites; late LEs, LAMP1- and cathepsin B/D-positive degradative lysosomes are enriched in the proximal regions of major dendrites and the soma. This also indicates that dendrites lack acidified lysosomes with active cathepsins and require a specific transport of cargo to the soma for degradation, whereas materials in the distal region of axons of mouse cortical neurons can be degraded locally by soma-derived degradative lysosomes that are constantly delivered to distal axons [40]. JIP3 has been reported to be a negative regulator of axonal lysosome abundance, and it functions as an essential adaptor protein localized to lysosomes in this part of APP transport. It has been shown by immunofluorescence labeling and electron microscopy that lysosomes increasingly accumulate in the axons of JIP3 KO mouse cortical neurons, suggesting that lysosomal transport defects might contribute to lysosome-filled axonal swellings at amyloid plaques [59]. Lysosomes within these axonal swellings contain low or undetectable levels of luminal proteases and move only locally with occasional possibilities of escaping from these restricted axonal swellings. This indicates that JIP3 is involved in retrograde transport and the maturation of distally formed lysosome precursors and thus, contributes to maintain a normal axonal lysosome distribution. Moreover, SorLA has been reported to be partially colocalized with endocytic compartments [51]; for example, SorLA stained by gold nanoparticles is frequently concentrated around MVBs in rat and human cortexes [30]; thus, SorLA can also be involved in this lysosomal pathway [51].

#### 5.2.4. Assisted Neuronal Transport of APP Species

APP species are firstly thought to be sorted into axons and dendrites based on specific neuronal signal sequences located at various domains. However, disruptions in multiple intracellular or extracellular domains of APP, including its YENPTY motif for internalization, do not affect its neuronal sorting and subcellular location [60].

**Figure 3:**
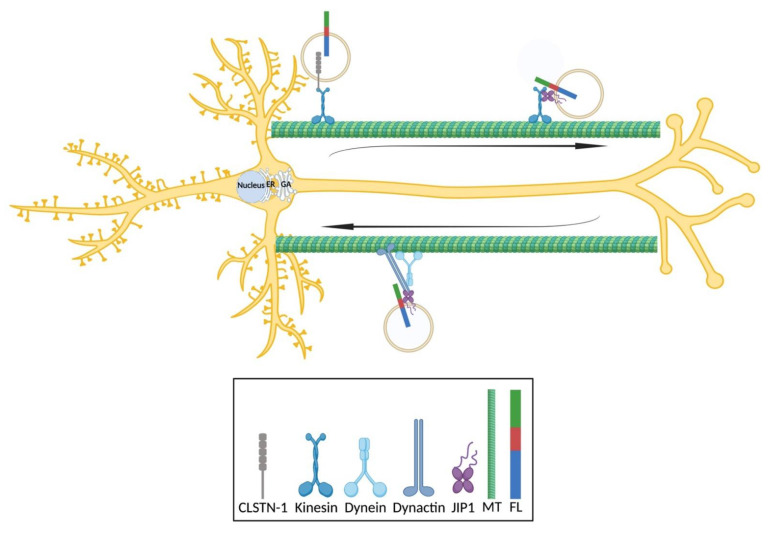
Trafficking of APP in neurons. In anterograde transport, APP is delivered by kinesin in a process including calsyntenin 1 or JIP1. In retrograde transport, APP is delivered by the dynein–dynactin complex with the assistance of JIP1. FL is short for full-length APP; MT is short for microtubule, and CLSTN-1 is short for Calsyntenin 1. This figure was produced with BioRender.com.

The short-distance transport of APP species within the somatic region includes those to and from the PM and those between various intracellular compartments. Long-distance transport, on the other hand, is between the soma and neurites. In both cases, APP transport relies mainly on the polarized organization of microtubules and its associated motor proteins: kinesin and dynein (Figure 3). APP anterograde transport depends mainly on kinesin-1 and its adaptor proteins, for example, JIP1 and calsyntenin 1. It has been reported by a molecular dynamics study based on protein structures that light chain-1 of kinesin-1 provides binding sites for both APP and JIP1 and can thus deliver APP directly or indirectly with JIP1 [61]. In support of this finding, the depletion of JIP1 with siRNA in mouse dorsal root ganglion neurons led to a striking decrease in the number of APP-positive vesicles and APP motility in both directions [43]. Interestingly, a reported mutation introduced at S460 of the kinesin light chain in rat cortical neurons also caused a reduction of axonal APP transport with regards to the percentage of motile APP-positive vesicles and velocity in both directions [62]. This could be an indication of disrupted binding between JIP1 and APP. Possibly, APP binds indirectly onto kinesin-1 through the JIP1b subunit in mixed mouse hippocampal and cortical neurons, and the depletion of JIP1 in JIP1-KO mice significantly impaired APP anterograde transport [63]. This could be due to a disrupted complex formation or the activation of kinesin-1. On the other hand, it has been shown that the silencing of JIP1 using siRNA in rat cortical neurons does not affect APP transport with regards to its velocity in both directions [64], which could be explained by the selected depletion of JIP1 subunits. By electron microscopy, it has been shown that calsyntenin 1 colocalizes with the TGN in the mouse brain [65]. Calsyntenin 1 binds to the kinesin light chain via its light chain-1 binding motifs and acts as an adaptor protein between APP and kinesin-1, which is supported by its colocalization with APP in axons and its predominantly anterograde co-transport with APP in the axons of rat cortical neurons [66]. The depletion of calsyntenin 1 by siRNA, on the other hand, remarkably disrupts the axonal transport of APP, increasing APP levels in the TGN of rat cortical neurons [66]. This could suggest that calsyntenin 1 mediates the exit of APP from the TGN and assists in the kinesin-mediated retrograde transport of APP in axons. However, the co-transport of calsyntenin 1 with APP in rat cortical neurons is also retrograde, suggesting that it might contribute to the dynein-mediated retrograde transport of APP in axons [66]. 

### 5.3. Distribution of APP Species

In neurons, APP is transported along secretory, endocytic and recycling routes, and its cleavages occur at multiple sites. After the removal of the signal peptide in the ER, APP species, including full-length APP and its processed products, can reach multiple intracellular compartments and be detected at the nucleus (AICD), the nuclear membrane, the ER (including ER–mitochondria contact sites), ER–Golgi intermediate compartments, the GA, the TGN, post-Golgi secretory vesicles, the PM, endosomes, lysosomes and autophagosomes. In some of these intracellular compartments, APP species reside to fulfill specific functions [67]. Obviously, the transport and processing of APP are intimately related, and current concepts suggest that APP moves between secretory compartments, the PM and endosomes, and its ability to enter or avoid certain intracellular compartments determines its processing fate. However, due to technical limitations, such as limited microscopy resolution, recognition of various fragments by many antibodies (Table 1) as well as poor specificity of some antibodies, it is generally difficult to conclude which APP species is detected. 

APP species localize at both axonal and somatodendritic compartments in mouse cortical neurons [60]. It has recently been shown, by using STED microscopy providing a resolution down to around 20–30 nm, that the C-terminal labeling of APP shows vesicle-like structures with enrichment at presynapses, while N-terminal labeling shows a distribution that appears like closely spaced vesicles distributed along filamentous tracks [68], suggesting that CTFs and N-terminal fragments (NTFs) use different transport mechanisms. The enrichment of CTFs at presynapses is supported by the results from a combination of stochastic optical resolution microscopy and STED microscopy, showing that γ-secretase that further cleaves CTFs is present at the neuronal synapses of mouse hippocampal neurons [69], as well as the enrichment of the product, Aβ42, in small vesicles of the presynapse [70]. By using STED microscopy, it has been shown that there is a clear separation between CTFs and NTFs within the same EE in mouse hippocampal neurons (Figure 2, orange arrows): CTFs are more concentrated at or close to the surface, while NTFs are more diffusely localized in the center [68]. This indicates that the majority of full-length APP is processed into CTFs or NTFs early during EE maturation in the soma, and EEs therefore function as sorting organelles for APP species by budding NTFs or CTFs into distinct transport vesicles in the soma and releasing them at different subcellular locations. Immunofluorescence labeling and immunogold electron microscopy revealed that CTFs are present in the synaptic vesicles of rat hippocampal neurons and are colocalized partially with synaptophysin [71]. This is supported by results from mouse hippocampal neurons imaged using STED microscopy showing that (1) CTFs colocalize only with synaptophysin but not with PSD95 [68] and that (2) Aβ is present in small vesicles at presynaptic compartments, among which some differ from those responsible for neurotransmitter release [70]. Importantly, full-length APP and/or its NTFs are not observed either at presynapses or postsynapses [68]. Since there is no colocalization between CTFs and NTFs, this could indicate that no or little full-length APP is present at presynapses.

Overall, the distribution of different APP species varies, and they might use distinct vesicle populations for their transport. Therefore, it is a complicated transport network for APP within a neuron: between the neuronal soma, axon and dendrites.

**Table 1:** Summarized table for anti-APP antibodies used in Section 5. The antibodies are shown in the order in which they appear in Section 5.

Anti-APP Antibody	Epitopes	References Used
22C11	66–81 of APP695	[65,68]
Y188	682–687 of APP695	[68,72]
Anti-APP	675–770 of APP770	[71]
AF1168	18–288 or 365–411 of APP770	[32]
6E10	1–16 of Aβ	[55]
C1/6.1	676–695 of APP695	[56]

## 6. Dysregulated Neuronal Transport of APP in AD

### 6.1. ER Stress in AD

ER stress [73], which can be caused by a number of factors (e.g., misfolded protein in the ER lumen), leads to the accumulation of APP in the ER and an activation of mitochondria- and ER-mediated cell-death pathways. It has been reported that there is an accumulation of misfolded proteins, an altered level of ER stress protein and thus, an activation of unfolded protein responses due to prolonged ER stress in AD brains [74,75], which could suggest that alterations in ER homeostasis might be implicated in the neurodegenerative events that characterize this disorder. 

### 6.2. Golgi Fragmentation in AD

The maturation, sorting, processing and transport of APP and its processing enzymes require the proper functioning of the GA. Golgi membranes form a unique stacked structure maintained by its structural proteins, for example, GRASP65, and the abnormal modifications of these proteins could cause the Golgi fragmentation that occurs in neurons of AD brains. It has been reported that Golgi fragmentation [76] itself could enhance vesicle budding from the Golgi membrane, accelerate APP transport and thus, elevate APP/Aβ levels around the site of fragmentation. The accumulation of Aβ in turn leads to the extensive phosphorylation of Golgi structural proteins by cyclin-dependent kinase-5 (cdk5) and thus, Golgi fragmentation, in the hippocampal neurons of mouse brains, resulting in a feedback loop that could result in further damage to neurons. However, this defect can be rescued by the expression of nonphosphorylated mutants of Golgi structural proteins or the inhibition of cdk5. The GA is thus suggested to be a potential drug target for AD treatment.

### 6.3. Endocytic and Autophagic Dysfunction in AD

Endosome–lysosomal and autophagic pathways are exquisitely involved in APP processing and transport and are thus critical for AD pathogenesis, and perturbances in these pathways are strongly associated with AD. For example, there is an accumulation of autophagic vesicle-derived LAMP1-positive vesicles containing low levels of lysosomal proteases within swollen neuronal axons at amyloid plaques [77]. The binding of APP to clathrin is decreased in AD patients [78]. Dynein dysfunction contributes to altered APP metabolism, resulting in the intracellular accumulation of APP and Aβ in monkey brains [79]. Disruptions in SorLA and its interacting adaptor proteins result in the disruption of APP transport and enhanced amyloidogenic processing [80,81]. Retromer deficiency is also observed in postmortem AD brains and a disruption in retromer-mediated endosomal recycling results in altered APP metabolism and the loss of cell surface receptors that are critical for neuronal plasticity and synaptic health and thus, AD pathology [82,83].

## 7. Discussion

Although it is clear that APP transport is crucial to the physiology and pathology of neurons, the underlying mechanisms behind APP transport pathways remain only partly understood. (1) Firstly, most previous studies only investigated certain part(s) of one or several pathway(s), whereas the rest of the networks remained uncontrolled. Considering the complexity of cellular transportation systems within neurons, including a large number of scaffolding, adaptor or effector proteins, regulatory signaling pathways and intracellular transport vesicles involved, this could result in incomplete conclusions. (2) Secondly, it is not easy to label APP species, and many reports without detailed information about accurate labeling make it even harder to conclude which APP species we are looking at. In most of the studies focusing on APP transport in neurons, there are two methods to label APP species: immunolabeling with anti-APP antibodies or tagged-APP constructs. Since APP is extensively proteolytically processed, it is uncertain whether each labeling reflects full-length APP and/or its processed products. Some of the previous studies using APP constructs containing no indication of where the fluorescent tag is attached makes it even harder to identify which APP species is visualized. Therefore, studies relying on only one antibody or label to detect APP provide results that are difficult to interpret. A solution to this problem is provided by an expression of dual-tagged APP constructs, with distinct tags introduced into both C- and N-terminal regions. However, the data can be misleading sometimes because of the steric hindrance or epitope masking by proteins bound to APP. This particularly applies to epitopes from its cytoplasmic domains, where many protein-interaction domains or ligand-binding sites reside. Some large fluorescent molecules, for example green fluorescent protein fused to the C-terminus, likely interfere with the binding of proteins that normally attach there. Thus, it is likely that tagged APP species will be transported within neurons in a manner that differs from that of endogenous APP. (3) Thirdly, anti-APP antibodies used in each study are sometimes different. For example, even for targeting CTFs, there are a number of commercial or homemade antibodies against different sequences: 643–695, 682–687, 687–695. All of these could provide slightly different results, for example, just for the localization of APP species. (4) Fourthly, the resolution of detection of the tag is another practical issue in the accurate interpretation of APP intracellular locations in neurons. In most of the studies, it is difficult to interpret when the resolution is a limiting factor. It varies among reports and usually shows fewer details. For example, two-channel imaging of a dual-tagged APP construct mostly shows the colocalization of two fluorescent proteins on C- and N-terminus of APP, with some separation. However, this colocalization can be interpreted as either the presence of full-length APP or a colocalization of NTFs and CTFs. Additionally, a colocalization of two signals (for example between APP and synaptophysin) is difficult to confirm when they are just near or on top of each other with limited resolution. To confirm such a result, it is necessary to identify axons and dendrites, followed by an identification of post- and presynapses. Another example is the over-processing of fluorescent signals. Although an enhanced signal after image processing possibly results in a broad distribution of APP species localized throughout the neuronal soma and neurites, a quantitative analysis could show that the intensity ratio of these two fluorescent tags varies largely between labeled structures in neurites. Hence, some useful information remains hidden unless the expression levels are kept low. (5) Last but not least, the (over)expression of a number of APP constructs are also used in some studies. One problem is that different fluorescent proteins or tags could give us distinct signals. For example, as discussed earlier, red fluorescent proteins are generally more stable in acidic compartments than green, and this results in a different distribution of APP species when interpreting with different constructs. Another potential problem is that overexpression itself could result in the activation of stress protocols and alternative mechanisms in neurons due to neurotoxicity, cell stress and cell death. Although many reports state that there is negligible difference between overexpressed and endogenous APP, this could still raise the question of whether the resulting transport of APP species might be distinct from that of endogenous APP. Overall, the cellular transport of APP itself is complicated enough to study, regardless of all the essential molecules, networks and pathways involved.

## 8. Conclusions

In conclusion, here we have reviewed neuronal APP transport and summarized critical mechanisms, including its essential lipoprotein receptors SorLA and sortilin and its essential adaptor proteins JIP1 and calsyntenin 1. In long-distance transport between the soma and neurites, APP species are transported within intracellular compartments with the assistance of the cytoskeleton, motor proteins and adaptor proteins (not only those discussed here). In anterograde direction from the soma, APP transport is mediated by kinesin directly or indirectly by forming a complex in the presence of calsyntenin 1 or JIP1. In retrograde direction towards the soma, APP transport is mediated by the dynein–dynactin complex, which also forms an indirect complex with JIP1. The retrograde transport of APP towards the soma is somewhat similar in dendrites and axons, sending out materials for degradation. However, APP transport in dendrites is overall far more complicated than in axons; for example, its bipolar microtubule arrangement results in a bidirectional, sometimes random or repeated, distribution of cargo-containing transport vesicles. In short-distance transport, mostly within the somatic region on the other hand, APP is trafficked along secretory, endocytic and recycling routes, where its cleavages occur in temporarily and spatially separated subcellular compartments. SorLA mainly functions between the TGN and EEs in assistance with adaptor proteins (not only those listed): GGAs in anterograde direction towards EEs and the retromer complex in retrograde direction towards the TGN. Sortilin, however, mainly functions in the endocytic pathway towards lysosomes. Moreover, we present here some major disruptions in the neuronal transport of APP that could contribute to AD pathology and discuss current methods and technical difficulties in the studies of APP transport, namely: (1) a study of overall picture of APP transport is generally impossible, (2) inefficient labeling of APP makes it difficult to identify or localize APP species, (3) a variety of anti-APP antibodies that label different epitopes could result in distinct interpretation, (4) a limited resolution for the detection of tagged APP species is not sufficient for a clear demonstration and (5) different tags or expression levels by themselves could provide distinct interpretation. In general, the neuronal trafficking of APP is complicated to study. In order to understand its mechanisms and defects better in the future, it is important to use more precise methodology. For example, we need more stable and less toxic tags that are able to target specific fragments of APP and thus, give us more accurate information about their distribution in neurons; we need super-resolution microscopes that can be used for the rapid imaging of live neurons that are able to discriminate signals more precisely in 3D and within even a few nanometers and thus, disclose their false positive colocalization with subcellular markers.

## Data Availability

Not Applicable.
